# Supplementary material for: Retaining public health volunteers beyond COVID-19
Source: PLoS One. 2023 Nov 13;18(11):e0294157. doi: 10.1371/journal.pone.0294157 (PMC10642819; doi:10.1371/journal.pone.0294157)
Supplement: S1 Appendix — (DOCX) [file pone.0294157.s001.docx]

**FOCUS GROUP DISCUSSION TOPIC guide**

**This is the starting topic guide. The overarching objectives will remain the same, but questions and prompts will be developed as focus group discussions are undertaken to incorporate any important themes that emerge.**

**Instructions:**

- Restate the purpose of the Focus Group Discussion – *To understand the experiences and role of COVID-19 Community Champions*
- Explain that you are there to understand more about their experiences and that they will have some time at the end of the interview to talk about any other issues that are important to them that may not have been covered by the questions.
- Check if participants have any questions
- Start audio-recording
- Begin the focus group discussion.

**Topics to be covered in the FOCUS GROUP DISCUSSION**

Why did you choose to be a COVID-19 Community Champion?

Did you have any doubts about becoming a Champion?

Were you given enough information?

Have you found Birmingham City Council (BCC) to be helpful in your role as a Champion?

Have they done anything particularly well or badly?

How could they improve?

Would peer support be helpful?

What do you do in your role as a Champion?

What do you do with the information BCC provides?

What would make your role easier/better?

Would you like to do more in your role as a Champion?

Did BCC share non-COVID content?

Would you like to do things unrelated to COVID?

How could you be supported to do this?

Would you recommend being a Champion to others?

Can you explain your choice?

Do you feel like you are appreciated by BCC as a Champion?

How can this be improved?
