# Supplementary material for: Retaining public health volunteers beyond COVID-19
Source: PLoS One. 2023 Nov 13;18(11):e0294157. doi: 10.1371/journal.pone.0294157 (PMC10642819; doi:10.1371/journal.pone.0294157)
Supplement: Attachment — Submitted filename: Point by point_Final (1).pdf [file pone.0294157.s006.pdf]

Dr Ameeta Retzer
Centre for Patient-Reported Outcomes Research & NIHR Applied Research Collaboration
Institute of Applied Health Research
University of Birmingham, B15 2TT
Phone: 0121 414 7866
Email: a.retzer@bham.ac.uk

27 April 2022

Dear Editor,

Thank you for the opportunity to engage in the peer review process in relation to this paper, “Retaining Public Health Volunteers beyond COVID-19” (PONE-D-21-37424R2). Both peer reviewers stated that our manuscript was well-written, focused on an important topic, and served as “an excellent example of informal, spontaneous volunteer engagement for the dissemination of public health information during a crisis”. Peer reviewer feedback requested the addition of some detail about the COVID champions volunteer programme, and some minor clarifications to the text. Neither reviewers raised any concerns about the methodological conduct of the study, stating that the manuscript was technically sound and the data supported the conclusions. The previous editor requested minor clarifications, which we provided.

Therefore, we were extremely disappointed to receive the most feedback from a new editor, and the decision to not proceed with publication. We believe the issues raised strongly contradict the judgment of the manuscript received to date from the peer reviewers and previous editor, thus we wish to formally appeal the decision. We present a point-by-point response to the recent comments below.

Editorial comment: The statistical analysis specifically approach of the Thematic analysis has not been performed appropriately and interpreted.

Thematic analysis is a form of qualitative study and is not a quantitative, statistical approach that aims for generalisability. It was undertaken in this research by two experienced qualitative researchers and reported transparently in accordance with the consolidated criteria for reporting qualitative research (COREQ) checklist[1]. The completed checklist has been submitted along with the manuscript as an appendix. The ‘rapid’ qualitative method reported in this manuscript pertained only to the use of field-notes rather than verbatim transcripts, alongside a pragmatic approach to participant recruitment, both of which were responses to the rapidly evolving context of the pandemic. This was to ensure the experiences captured from participants were comparable and the findings were not outdated. Rapid qualitative methods have been found to be methodologically sound[2], all data were analysed and interpreted following standard and accepted methods of thematic analysis[3], and our approach is outlined in detail in the manuscript.

Editorial comment: The issues discussed in the paper are exciting but lacks appropriate analysis and have significant demographic variations in the

sample. The interpretations are not in line with addressing the large deviations in the sample.

Sample size calculations are not used in qualitative research, instead recruitment and sample size are guided by the concept of data saturation. This is widely used in qualitative research and refers to the point at which no new information is discovered in data analysis[4]. This is reached through a deliberate effort to recruit participants across a wide range of demographic characteristics. This is associated with the premise of qualitative research being to capture and understand the breadth and diversity of experience rather than to quantify those experiences in a statistical sense. The interpretations reported are directly from these data and a robust approach was used through mechanisms such as double-coding, as per accepted qualitative practice[5].

Editorial comment: Additionally, the research lacks the presence of any conceptual framework or hypothesis the authors are trying to prove.

The lack of a conceptual framework/hypothesis is because the approach used was largely inductive rather than deductive, i.e. we allowed the data to determine the themes as we did not have pre-conceived themes we expected to find. However, we cite the use of the Gaskin model of volunteer involvement to inform our understanding of the topic and to formulate the initial coding frame in addition to the research aims[6]. A flexible iterative approach was used to allow for the exploration of novel themes. This is an accepted and widely used approach to explore topics with limited evidence base and diversity of experiences[7].

We respectfully request that the decision to reject this manuscript is reconsidered in light of the information we have provided. We believe this topic is of interest, the methods used are robust and have been reported transparently.

Thank you for considering this appeal.

Yours sincerely,

Ameeta Retzer

References

1. Tong, A., P. Sainsbury, and J. Craig, *Consolidated criteria for reporting qualitative research (COREQ): a 32-item checklist for interviews and focus groups.* Int J Qual Health Care, 2007. **19**(6): p. 349-57.
2. Gale, R.C., et al., *Comparison of rapid vs in-depth qualitative analytic methods from a process evaluation of academic detailing in the Veterans Health Administration.* Implementation Science, 2019. **14**(1): p. 11.
3. Hsieh, H.F. and S.E. Shannon, *Three approaches to qualitative content analysis.* Qual Health Res, 2005. **15**(9): p. 1277-88.
4. Saunders, B. and J. Sim, *Saturation in qualitative research: exploring its conceptualization and operationalization.* 2018. **52**(4): p. 1893-1907.
5. O'Connor, C. and H. Joffe, *Intercoder Reliability in Qualitative Research: Debates and Practical Guidelines.* International Journal of Qualitative Methods, 2020. **19**: p. 160940691989922.
6. Gaskin, K. *A choice blend: what volunteers want from organisation and management*. 2003 [cited 2022 April 27].
7. Fereday, J. and E. Muir-Cochrane, *Demonstrating Rigor Using Thematic Analysis: A Hybrid Approach of Inductive and Deductive Coding and Theme Development.* International Journal of Qualitative Methods, 2006. **5**: p. 80 - 92.
