# Supplementary material for: Retaining public health volunteers beyond COVID-19
Source: PLoS One. 2023 Nov 13;18(11):e0294157. doi: 10.1371/journal.pone.0294157 (PMC10642819; doi:10.1371/journal.pone.0294157)
Supplement: Attachment — Submitted filename: Response to reviewer - point by point 180222.docx [file pone.0294157.s005.docx]

| Comment | Author response |
| --- | --- |
| Editor Comments |  |
| Please ensure that your manuscript meets PLOS ONE's style requirements, including those for file naming. The PLOS ONE style templates can be found at  https://journals.plos.org/plosone/s/file?id=wjVg/PLOSOne_formatting_sample_main_body.pdf and  https://journals.plos.org/plosone/s/file?id=ba62/PLOSOne_formatting_sample_title_authors_affiliations.pdf. | Thank you, the manuscript has now been re-formatted to include Heading Level 1 for the main titles, the appendix has been re-titled to Fig 1, and the figure legend has been added underneath the references. The title page has also been amended as per the author affiliation guidance. |
| Please provide additional details regarding participant consent. In the ethics statement in the Methods and online submission information, please ensure that you have specified whether:  i) whether the ethics committee approved the verbal/oral consent procedure, ii) why written consent could not be obtained, iii) how verbal/oral consent was recorded, If the need for consent was waived by the ethics committee, please include this information. | Thank you. The ethics statement has now been edited to show that the ethics committee approved the study and all its procedures. Explanation for use of verbal consent instead of written has been added, as has how verbal consent was recorded. That the participants were all adults has now been added to the recruitment section. The need for consent was not waived. |
| Thank you for stating the following in the Acknowledgments Section of your manuscript:  [We would like to thank the National Institute for Health Research ARCWM Public Health Theme Patient and Public Involvement group for their invaluable contributions. This study was funded by the National Institute for Health Research (NIHR) Applied Health Research Collaboration West Midlands. The views expressed in this article are those of the author(s) and not necessarily those of the University of Birmingham, Nation Health Service (NHS), National Institute for Health Research (NIHR) or the Department of Health and Social Care.]  We note that you have provided funding information that is not currently declared in your Funding Statement. However, funding information should not appear in the Acknowledgments section or other areas of your manuscript. We will only publish funding information present in the Funding Statement section of the online submission form.  Please remove any funding-related text from the manuscript and let us know how you would like to update your Funding Statement. Currently, your Funding Statement reads as follows:  [This study was funded by the National Institute for Health Research (NIHR) Applied Health Research Collaboration West Midlands.]  Please include your amended statements within your cover letter; we will change the online submission form on your behalf. | Thank you, this statement has been removed and we will request that the funder contribution statement is also added to the online form. |
| Additional Editor Comments |  |
| I recommend that the authors summarize the findings in one figure or table in the main body of the paper; the Appendix is well organized, but it would be preferable to include the concise figure (or table) in the main body of the paper so that the reader can grasp the findings of this paper at a glance.  Please explain what the authors mean "FGD A" etc. in the Appendix. | Thank you, the acronym has now been explained underneath Table 1. The contents of this appendix have now been integrated within the results section of the text, as per the request by Reviewer 2. Please could we request an editorial steer regarding how to manage these contradictory comments? We have integrated quotes within the text but have tried to promote readability and minimise word-count by using only key quotes. In addition to this, if the full range of quotes is preferred, we could still submit the manuscript with the original appendix. |
| Reviewer 1 Comment |  |
| Changes in people's volunteering behaviors by the COVID-19 pandemic is an important topic and the manuscript was well written.  However, data collection was made among COVID-19 related volunteers during COVID-19 pandemic and this may make the findings difficult to be generalized, compared to data collection among volunteers engaging other public health issues.  It has been said that the data collection were made until data saturation was reached, however, is there any gender or age specific differences in volunteering motives? | Thank you. There were not enough people to explore these differences, however almost one third of the people involved in the study were retired. A statement around the limitations of the timings of the interviews/group discussions has been added to reflect this. |
| Reviewer 2 Comment |  |
| **Abstract**  Line 58 Define the acronym of NHS for a more global audience (like you do in Acknowledgements). | Thank you, this has been edited. |
| Line 60 There is an extra space between “reported experiences” and “to consider the potential for volunteer retention…” | Thank you, this has been edited. |
| Lines 66-67 The double use of ‘using’ in this sentence doesn’t read well. See if you can come up with another synonym. | Thank you, this has been edited to “analysed through…” |
| Line 78 Please edit the double period. | Thank you, this has been edited |
| **1. Introduction**  The first paragraph nicely establishes how this work is in support of the UK Nation Health Service’s long-term plan of transitioning towards a “participation culture.” “…such as the NHS Volunteer Responder scheme…” Please again define NHS here for the remaining body of the manuscript. There is a space between the end of the sentence and citation [2], but otherwise all citations are conveyed without a space. Please make consistent throughout. | Thank you, NHS has been defined and the citation spacing corrected. |
| Please delete “However,” at the start of your topic sentence for paragraph two and simply start with “The most effective way…” Topic sentences should not begin with a transitional phrase. | Thank you, this has been removed. |
| Because the Gaskin’s model of volunteer involvement was used to help develop an initial coding framework for your qualitative data analysis, I am interested in knowing more about this framework in your Introduction. Please consider some expansion about this model in a separate small paragraph of your Introduction. | Thank you, this section has been expanded. |
| Box 1 Do not use the acronym PPE here (just write out personal protective equipment) or define the acronym PPE versus assume the reader is aware what it means. | Thank you, the full term has been added. |
| **2. Methods**  Should your appendices be listed consecutively? Appendix 1 for your reporting guidelines (here in Methods) and Appendix 2 for your illustrative verbatim quotations (next in Results)? | Thank you, Appendix 2 is now Appendix 1, and Appendix 2 has been removed as the quotes are included in the Results section. |
| Nice description of the pragmatic approach to recruitment. | Thank you. |
| Under data collection, why is it relevant to mention that the health researchers were female? – | Thank you, this is a reporting requirement from COREQ checklist to identify potential sources of bias. |
| **3. Results**  When describing how the study’s participants shared information, it is not clear what is meant by “telephone offline group individually.” Consider simplifying and stating as, “check in via telephone with individuals in the group to ensure they had the correct information.” Yes? | Thank you – this has now been simplified. |
| The narrative expansion of the six different themes was very clear. Under “Volunteer motivations and expectations,” consider adding the detail in parentheses on employment status to Box 1. | Thank you, an employment status section has now been added to Table 1. |
| Under “Volunteer management,” when referencing “this was especially pertinent in cases where the participant was sharing information with members of vulnerable groups who were unable to seek information themselves.” Can more information on the specific vulnerable groups be shared without breaching any confidentiality concerns? | Thank you, more information has been added about the groups the individual was working with and an illustrative quote has been included. |
| Please capitalize the proper noun “council” in the sentence “For example, some participants described simply forwarding the emails they had received from the Council…” As a reader, I found myself going back to the Methods to see what was meant by council. In the Methods you reference the Birmingham City Council Public Health Team, but later shorten your reference to this group as “members of the Public Health team” and “the council.” Please consider keeping your shortened reference to this group consistent throughout the paper for ease of understanding. | Thank you, this has been edited throughout the paper for consistency. |
| Excellent job exploring the potentially missed opportunity of pooling volunteers’ skills. | Thank you. |
| Under “Feeling valued,” similar to the comment about vulnerable groups under the “Volunteer management” theme, I find myself curious about the participants who received a lot of appreciation from their target group. Which target groups were more appreciative? This may be helpful information with advancing the use of Community Champions for other public health issues beyond the pandemic. | Thank you, more detail about this has been added. |
| Under “Continued need for the role,” the statement “and there was less of the initial urgency seen when the programme started” is redundant. Possibly, better to write “and there was less urgency” noted or observed “compared to when the programme started.” | Thank you, this has now been edited. |
| Please excuse the following comments as they likely relate to this reviewer’s American English versus the writers presumed British English. Under “Continued need for the role,” in the sentence “Some discussed their interest in expanding their role to other efforts countering COVID-19.” Countering? Meaning going against? This is not clear. Possibly consider “beyond” instead of “countering.” As well, in the sentence, “Many stated that their express permission would be required…” This reader inferred what was meant by “their express permission” by the context of the remaining sentences in this paragraph. Possibly consider removing “express” to minimize confusion. | Thank you, the word “countering” has now been removed for clarity. |
| Under “Interest in new responsibilities,” as the reader, I appreciated learning about the comment that the programme would benefit from an expenses reimbursement policy.” When reading that a few volunteers were using personal funds under the “Volunteer management” theme earlier in the Results, this reviewer was hoping the sustainability of using personal funds would be questioned and addressed elsewhere. | Thank you, this has now been mentioned in the Discussion section. |
| The themes and illustrative verbatim quotations may have been sequestered to the appendix because of the limitations of word count. If not, as a reader, I would prefer they be embedded, respectively, throughout the narrative expansion of the six different themes in the text. | Thank you. The quotes in Appendix 2 have now been distributed throughout the results section and this appendix has been removed. The addition of these quotes in the main body has increased the length considerably. I hope this is alright. |
| **4. Discussion**  I enjoyed reading about your exploration of the identified importance of participant permission should the programme’s expectation evolve over time, as well as, the potentially missed opportunity of how to pool volunteers’ skills. Thank you for addressing the importance of supplementing the participants’ out of pocket expenses again here in the Discussion. | Thank you. |
| I might suggest better connecting and inserting some of the defined language from the Abstract and Introduction, like informal, formal, spontaneous volunteer engagement into the Discussion here… how to convert informal spontaneous volunteers into more formal volunteers for the long-term, so the paper reads seamlessly. | Thank you, text relating to formal/informal volunteering and spontaneous volunteering has now been added to the first part of the discussion. |
| You discuss the focus group methodology in the Discussion here, but in your Methods, you do not explicitly state which 11 participants (using identifying characteristics) partook in the three conducted focus groups. Were the majority of participants in the focus groups White British? If appropriate, go back to the Methods and be more transparent about which participants comprised your focus groups as this will help to further substantiate your discussion points about the generated dynamic group interaction. | Thank you. Table 1 has now been edited to include the demographic breakdown of the focus groups and interviews |
| Nice job placing a positive spin on your use of rapid qualitative data analysis Methods and sharing ideas for future research. | Thank you! |
| Please add citation(s) to the following statement, “… of these findings beyond the COVID-19 context is evidenced by their resonance with the current literature.” | Thank you, these have been added. |
| Would the last paragraph referencing the PPI group be better conveyed in Acknowledgements? Seems like a last minute add-on or after thought the way it currently conveys in the Discussion. Discussion might be better concluded with second to last paragraph anyway. If not appropriate for the Acknowledgements, this information on the PPI group would be better placed at the beginning of the second to last paragraph (the paragraph on the study’s rigorous methodology). | Thank you, this section has now been moved to the beginning of the second to last paragraph. |
| Spacing of the within text citations, like in the Introduction, is slightly off in the Discussion, as well. | Thank you, this has been edited. |
| The first sentence under “Conclusions” may be better stated as, “Our aim was to explore the views and experiences of individuals involved in public health volunteering during the COVID-19 pandemic, as well as, to identify factors relating to retention, recruitment, and expansion of their roles.” Nice succinct summary sentence of the study’s findings. | Thank you! |
