# Supplementary material for: Retaining public health volunteers beyond COVID-19
Source: PLoS One. 2023 Nov 13;18(11):e0294157. doi: 10.1371/journal.pone.0294157 (PMC10642819; doi:10.1371/journal.pone.0294157)
Supplement: Attachment — Submitted filename: COVIDChampions_R2R_29Sept.docx [file pone.0294157.s007.docx]

| Reviewer comment | Author response |
| --- | --- |
| Reviewer #4: Explain in the methodology part how many participants involved in semi-structured focus group discussions, one-to-one interviews and email interviews | Thank you – this has been reported in the results section, broken down by focus group discussions, interviews, and email interview. |
| Reviewer #5: Dear author  Your article addresses an important issue regarding volunteer work.  Some observations and suggestions to your article:  1. Qualitative data collection was carried out using different methods, but they were analyzed and computed in the same way. The collection from a focus group brings a very different result from the collection by individual interview. In the focus group, the opinions expressed are influenced by the interaction of the participants, so I would not recommend the use of different methods for this issue.  2. I would include in the methods which were the guiding topics of the focus group and which were the questions of the individual interviews.  3. I would be cautious in generalizing the results to other cultures. | Thank you for this feedback.  1. The decision to undertake both interviews and focus group discussions was a pragmatic one intended to accommodate as many people as possible in the rapid evaluation. We have included the combination of the two forms of data as a limitation in our discussion (p15) “This also meant a range of different qualitative data was collected and analysed together, which must be considered.”  2. The topic guide has now been added as an appendix and a statement has been added to the methods section to clarify that the same topic guide was used for each (p5) “The same topic guide was used for the focus groups, one-to-one interviews and email interviews (Appendix 1).”  3. Thank you – this has now been explicitly stated in the discussion (p15) “However, the applicability of these findings to other cultures would need to be interrogated as the programme took place in and participants were recruited from the UK, a high-income country.” |
| Reviewer #6: This is a well-written manuscript.  The study objective is clear and the methods used are appropriate and well detailed.  In my opinion, the revisions made to issues made by previous reviewers are sufficient.  I recommend for acceptance. | Thank you for this feedback. |
